# Targeting GPR84 to alleviate acute immune-mediated liver injury

**DOI:** 10.1186/s10020-025-01248-9

**Published:** 2025-05-14

**Authors:** Yanan Zheng, Yumeng Wang, Yujie Xu, Shanshan Shen, Haozhe Xu, Chao Hu, Yongzhen Chen, Fengmeng Teng, Jinshun Pan, Shuqian Zheng, Junqi Wang, Zhongping Su, Qiang You

**Affiliations:** 1 Department of Geriatrics, Department of Biotherapy, the Second Affiliated Hospital of Nanjing Medical University, Nanjing Medical University, Nanjing, 210011 China; 2 Department of Surgery, the Fourth Affiliated Hospital of Nanjing Medical University, Nanjing, 210031 China; 3 Jinhua Hospital Affiliated to Zhejiang University School of Medicine, Jinhua, 321000 China; 4 Department of General Practice, the Second Affiliated Hospital of Nanjing Medical University, Nanjing, 210011 China; 5 Affilated Hospital of Nanjing University of Chinese Medicine, Nanjing, 210029 China; 6 Shenzhen Traditional Chinese Medicine Hospital, Shenzhen, 518033 China; 7 Department of Geriatric Gastroenterology, Neuroendocrine Tumor Center, Jiangsu Province Hospital, the First Affiliated Hospital of Nanjing Medical University, Institute of Neuroendocrine Tumor, Nanjing Medical University, Nanjing, 210029 China

**Keywords:** GPR84, Immune-mediated liver injury, Inflammation, GLPG1205

## Abstract

**Background:**

GPR84 is a Gi-coupled G-protein-coupled receptor (GPCR) predominantly expressed in immune cells, with its expression upregulated during inflammatory conditions. However, its specific role in immune-mediated liver injury remains unclear.

**Methods:**

We utilized a concanavalin A (Con A)-induced mouse model to simulate immune-mediated liver injury. The expression of GPR84 was assessed by quantitative RT-PCR and western blotting. GPR84 gene knockout mice were employed to evaluate the receptor’s functional role. Bone marrow chimeric mice were created to determine the involvement of hematopoietic cells. Infiltrating liver inflammatory cells were analyzed by flow cytometry. The activation of key signaling pathways in hepatic tissues was assessed by western blotting. The GPR84 antagonist GLPG1205 was tested in this model to evaluate its therapeutic potential.

**Results:**

GPR84 expression was significantly upregulated in the mouse liver following Con A injection. Mice lacking GPR84 exhibited reduced serum ALT and AST levels, diminished liver damage, and decreased apoptosis. Additionally, the expression levels of inflammatory cytokines MCP-1 and TNF-α were significantly lower in *Gpr84*^−/−^ mice compared to wild-type (WT) mice after Con A injection. Flow cytometry analysis revealed a notable reduction in the proportion of Kupffer cells and infiltrating monocytes (CD11b⁺Ly6C^low^Ly6G⁻) in *Gpr84*^−/−^ mice. Using bone marrow chimeric mice, we demonstrated that GPR84-deficient bone marrow-derived cells mitigate Con A-induced liver injury. Furthermore, GPR84 deficiency was associated with reduced hepatic apoptosis and lower phosphorylation levels of STAT3, ERK, JNK, p38, and p65, effectively inhibiting key inflammatory signaling pathways. Importantly, treatment with the GPR84 antagonist GLPG1205 significantly lowered serum ALT and AST levels, reduced the expression of inflammatory cytokines, and alleviated liver damage.

**Conclusions:**

Our findings suggest that GPR84 plays a pivotal role in immune-mediated liver injury, primarily through its expression on hematopoietic cells. Targeting GPR84, particularly with the antagonist GLPG1205, offers a promising therapeutic strategy for treating immune-related liver diseases.

**Supplementary Information:**

The online version contains supplementary material available at 10.1186/s10020-025-01248-9.

## Introduction

GPR84 is an orphan class A G protein-coupled receptor (GPCR) that is associated with inflammation (Wittenberger et al. 2001; Yousefi et al. 2001). It is widely expressed across various human tissues, with particularly high expression in myeloid-derived cells, including monocytes, macrophages, neutrophils, and microglia (Lattin et al. 2008). It has also been shown that GPR84 is highly expressed in skeletal muscle, and its absence has been linked to detrimental effects on mitochondrial function (Montgomery et al. 2019). Studies have identified GPR84 as a receptor for medium-chain fatty acids (Wang et al. 2006), which modulates macrophage activation and suppresses excessive immune responses triggered by lipotoxicity. Consequently, GPR84 is regarded as a potential therapeutic target for a variety of diseases, including ulcerative colitis, fibrotic diseases, nonalcoholic steatohepatitis, and acute respiratory distress syndrome (Liu et al. 2023a). Several GPR84 agonists have been identified, including 6-n-octylaminouracil (6-OAU), 6-hexylamino-2,4(1H,3H)-pyrimidinedione (PSB-1584), diindolylmethane and its derivatives, as well as embelin and 2-(hexylthio)pyrimidine-4,6-diol (ZQ-16) (Wojciechowicz and Ma'ayan 2020). Three GPR84 antagonists—(S)−2-((1,4-dioxan-2-yl)methoxy)−9-(cyclopropylethynyl)−6,7-dihydro-4H-pyrimido[6,1-a]isoquinolin-4-one (GLPG1205), sodium 2-(3-pentylphenyl)acetate (PBI-4050), and sodium 2-(3,5-dipentylphenyl)acetate (PBI-4547)—have shown therapeutic effects in animal models of various inflammatory and fibrotic diseases (Chen et al. 2020). GPR84 has been identified as a pro-inflammatory receptor (Suzuki et al. 2013) that mediates myeloid cell infiltration (Puengel et al. 2020). The activation of the GPR84 receptor enhances the expression of phosphorylated Akt, p-ERK, and promotes the nuclear translocation of p65 under inflammatory conditions. Additionally, it increases the expression levels of various inflammatory mediators, including TNFα, IL-6, IL-12B, CCL2, CCL5, and CXCL1 (Recio et al. 2018). While the role of GPR84 in inflammation, fibrosis, and metabolism has been identified, the contribution of GPR84 to immune-mediated hepatic injury remains unexplored.

Concanavalin A (Con A)-induced liver injury is a well-established murine model used to study T-cell mediated hepatitis (Tiegs et al. 1992). When Con A is injected intravenously, it triggers acute liver injury and systemic immune activation in mice. T cells, natural killer (NK) cells, natural killer T (NKT) cells, and macrophages can induce hepatocyte cell death through direct cell-to-cell contact or by secreting proinflammatory cytokines like tumor necrosis factor alpha (TNF-α), interferon gamma (IFN-γ), interleukin (IL)−2, IL-6, and granulocyte–macrophage colony-stimulating factor, as well as reactive oxygen species (Wang et al. 2012). This model closely mimics the immune-mediated hepatitis pathology observed in humans and has been widely recognized and employed in studies related to immune hepatatis (He et al. 2017; Heymann et al. 2015; Zheng et al. 2020). The role of GPR84 involvement in the pathogenesis of Con A-induced immune liver injury remains undefined.

This study aims to elucidate the role of GPR84 within the context of the Con A-induced liver injury model. The results reveal that GPR84 deficiency significantly mitigates liver injury in mice subjected to Con A administration. Furthermore, pharmacological inhibition of GPR84 using the antagonist GLPG1205 demonstrates a pronounced therapeutic effect in this model. These findings suggest that GPR84 represents a potential therapeutic target for the treatment of immun liver injury, providing new insights into the development of novel interventions.

## Results

### GPR84 expression is upregulated in Con A-induced immune liver injury in mice

**Fig. 1:**
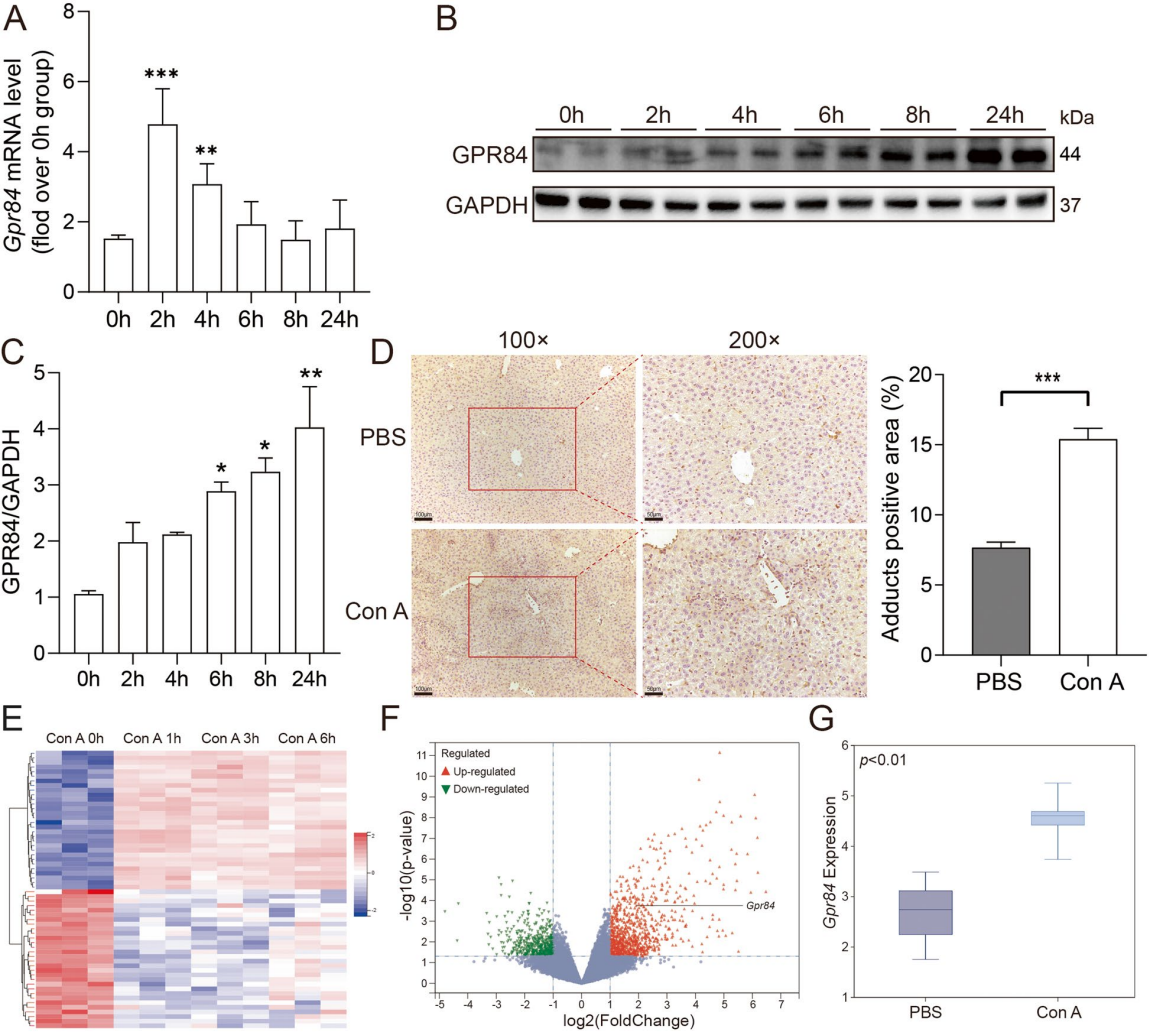
Elevated GPR84 expression in Con A-induced immune liver injury. Wild-type (WT) mice were injected with PBS or Con A (15 mg/kg) at 0 h, 2 h, 4 h, 6 h, 8 h, and 24 h. **A** qRT-PCR and (**B**, **C**) Western blot analysis showed increased GPR84 levels in liver tissue over time. **D** Representative immunohistochemical staining of GPR84 in liver sections after 24 h of treatment with PBS or Con A. **E**, **F** Heatmap and volcano plot of differentially expressed genes (DEGs) identified from the GSE17184 dataset, comparing Gpr84 expression in Con A-induced liver injury versus controls, with DEGs defined as |log2 fold change (FC)|> 1.0 and *P* < 0.05. **G** Box plot illustrating GPR84 expression levels in Con A-induced liver injury. Mean ± SEM. *P < 0.05, **P < 0.01, *** *P* < 0.001

To investigate the expression of GPR84 in immune liver injury, we first established a Con A-induced mouse model and examined *Gpr84* mRNA levels in liver tissues at various time points. The results showed an increase in hepatic *Gpr84* mRNA expression, peaking at 2 h post-injection (Fig. 1A). Correspondingly, *GPR84* protein levels displayed a time-dependent increase (Fig. 1B, C). In addition, immunohistochemical staining revealed that 24 h after tail vein injection of Con A, the expression of GPR84 protein was significantly elevated in liver tissues (Fig. 1D). To further validate these findings, we analyzed the GSE17184 dataset from the GEO database, comparing Gpr84 expression between Con A-induced liver injury and normal controls. The dataset confirmed that *Gpr84* mRNA expression was significantly upregulated in the Con A-induced liver injury model (Fig. 1G). These results were systematically visualized through heatmaps and volcano plots, highlighting the position of *Gpr84* among the differentially expressed genes (DEGs) (Fig. 1E, F). DEGs were identified using the criteria of |log2 fold change (FC)|> 1.0 and p-value < 0.05. Collectively, these findings indicate that GPR84 expression is markedly upregulated during the progression of Con A-induced immune liver injury in mice.

### GPR84 deficiency attenuates Con A-induced immune liver injury in mice

**Fig. 2:**
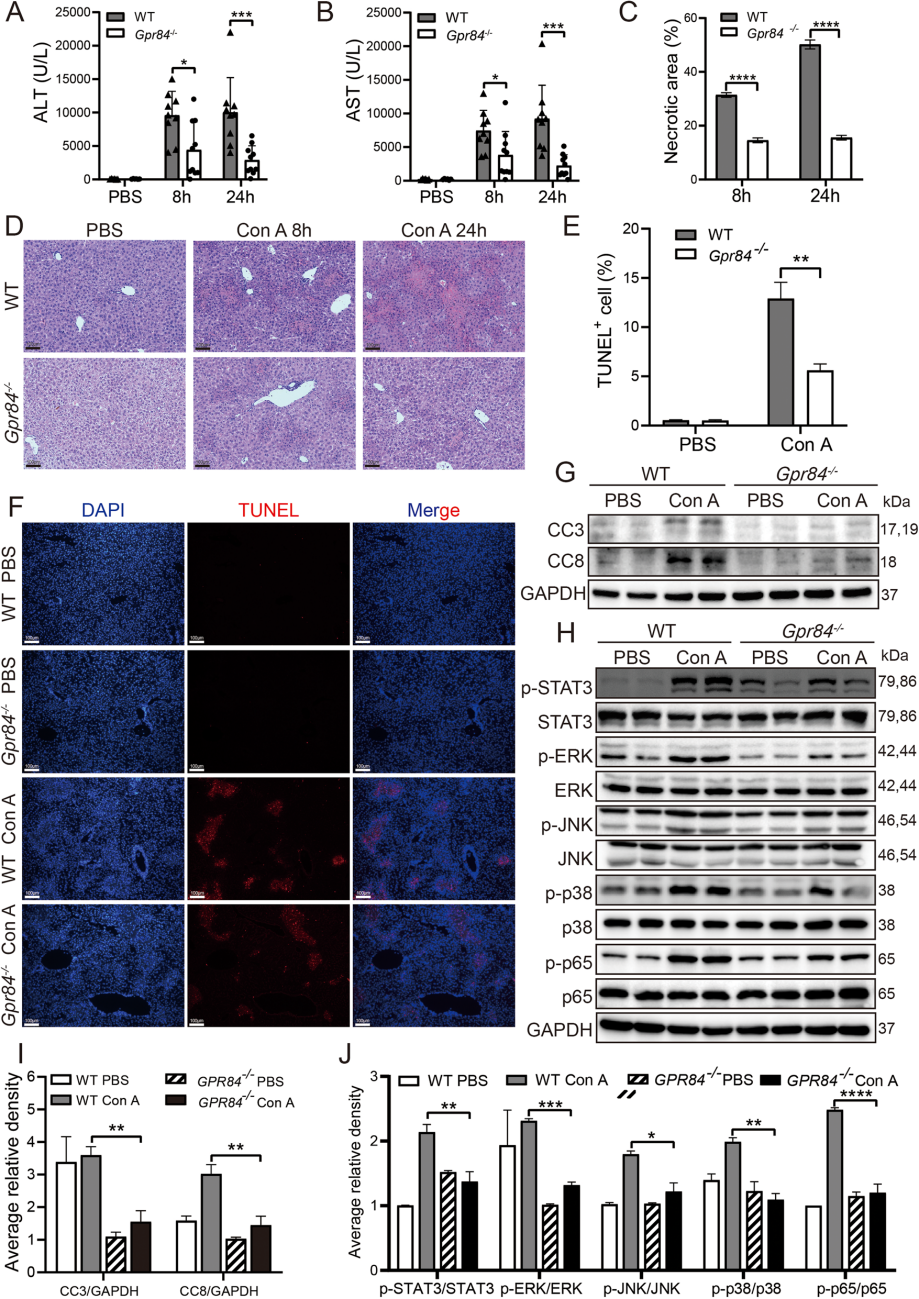
GPR84 deficiency alleviates Con A-induced liver injury. **A**, **B** Serum ALT and AST levels at 8 h and 24 h post-injection of PBS or Con A in WT (*n* = 9) and *Gpr84*^*−/−*^ (*n* = 10) mice. **C**, **D** Representative H&E staining of liver tissue, with necrotic areas quantified using ImageJ. **E**, **F** TUNEL staining of liver sections after 24 h treatment with PBS or Con A, with images captured at 100 μm scale. **G**, **I** Western blot analysis of Caspase-3 and Caspase-8 expression in liver tissue. **H**, **J** Western blot analysis of STAT3, MAPK, and NF-κB pathway proteins. Mean ± SEM. *P < 0.05, **P < 0.01, *** *P* < 0.001, **** *P* < 0.0001

To further explore the potential role of GPR84 in Con A-induced immune liver injury, we successfully generated Gpr84 knockout (*Gpr84*^*−/−*^) mice using CRISPR/Cas9 gene-editing technology (Fig. S1 A, B, C). We then established Con A-induced liver injury models in both wild-type (WT) and *Gpr84*^*−/−*^ mice. The results showed that, compared to WT mice, serum alanine transaminase (ALT) and aspartate transaminase (AST) levels were significantly reduced in *Gpr84*^*−/−*^ mice at 8 and 24 h post-Con A injection (Fig. 2A, B). Hematoxylin and eosin (H&E) staining further confirmed that the extent of liver damage in *Gpr84*^*−/−*^ mice was markedly smaller than that in WT mice (Fig. 2C, D). Additionally, TUNEL staining revealed a significantly lower proportion of apoptotic cells in the liver tissues of *Gpr84*^*−/−*^ mice compared to WT mice (Fig. 2E, F). To investigate changes in apoptosis-related pathways, we examined the expression levels of cleaved caspase-3 and caspase-8. The results indicated that the expression of both apoptotic proteins was significantly lower in the liver tissues of *Gpr84*^*−/−*^ mice following Con A administration, compared to WT mice (Fig. 2G, I). Previous studies have demonstrated that Con A-induced immune liver injury is closely linked to several inflammation-related signaling pathways, including IL6/JAK2/STAT3, JNK/p-JNK, p38 MAPK, and NF-κB (Zhou et al. 2022; Li et al. 2015; Liu et al. 2023b; Mo et al. 2018). To explore the involvement of these pathways, we analyzed the activation levels of key signaling molecules. The results showed that the phosphorylation levels of STAT3, ERK, JNK, p38, and p65 were significantly higher in WT mice compared to *Gpr84*^−/−^ mice following Con A administration (Fig. 2H, J). These findings demonstrate that GPR84 deficiency effectively attenuates Con A-induced immune liver injury by reducing apoptosis and suppressing the activation of inflammatory pathways, including STAT3, MAPK, and NF-κB, thus exerting a protective effect.

### GPR84 deficiency alters inflammatory cell infiltration in mice

**Fig. 3:**
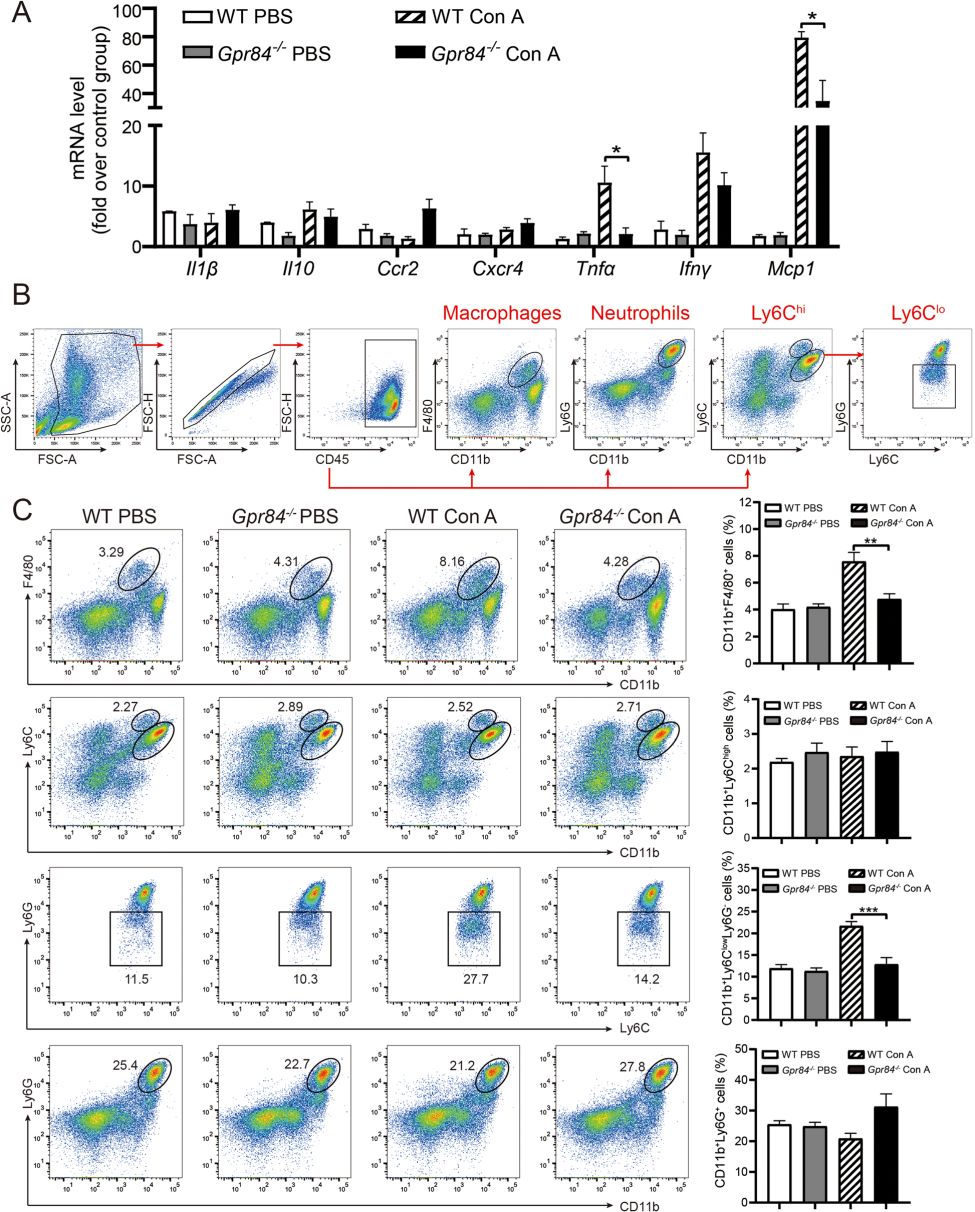
GPR84 deficiency alters immune cell infiltration in liver tissue. **A** qRT-PCR analysis of inflammatory cytokine expression in WT and *Gpr84*^*−/−*^ mice 24 h after PBS or Con A injection. **B** Fluorescence-activated cell sorting (FACS) gating strategy for immune cell analysis. **C** Flow cytometry analysis of non-parenchymal cells (NPCs) isolated from the liver of WT and *Gpr84*^*−/−*^ mice treated with PBS or Con A. Mean ± SEM. *P < 0.05, **P < 0.01, *** *P* < 0.001

To further clarify the specific impact of GPR84 deficiency on the inflammatory response, we investigated the changes in immune cell infiltration. qPCR analysis revealed that following Con A injection, the expression levels of the inflammatory cytokines *Mcp1* and *Tnfα* were significantly lower in *Gpr84*^*−/−*^ mice compared to WT mice (Fig. 3A), suggesting that GPR84 deficiency may attenuate liver inflammation. These findings indicate that GPR84 deficiency effectively alleviates the inflammatory response induced by Con A. We then performed flow cytometry to assess immune cell infiltration following Con A administration. The gating strategy for immune cell populations is shown in Fig. 3B and Supplementary Fig. S1E. The results demonstrated that 24 h post-Con A injection, the proportion of Kupffer cells (CD11b⁺F4/80⁺) and CD11b^+^Ly6 C^low^Ly6G^−^ infiltrating monocytes was significantly lower in *Gpr84*^*−/−*^ mice than in WT mice (Fig. 3C). However, no significant differences were observed in the proportions of neutrophils (CD11b⁺Ly6G⁺) or CD11b^+^Ly6 C^high^ infiltrating monocytes between the two groups (Fig. 3C). In addition, lymphocyte analysis revealed no significant differences in the proportions of CD4⁺ and CD8⁺ T cells between *Gpr84*^*−/−*^ and WT mice (Fig. S1F), which contrasts with previous reports suggesting that the Con A-induced model affects T cell infiltration (Ye et al. 2018). These findings suggest that GPR84 deficiency reduces the infiltration of macrophages and infiltrating monocytes (IMs) in the liver following Con A administration, without affecting T cell infiltration.

### GPR84 expression on hematopoietic cells drives ConA-induced cell death in the liver

**Fig. 4:**
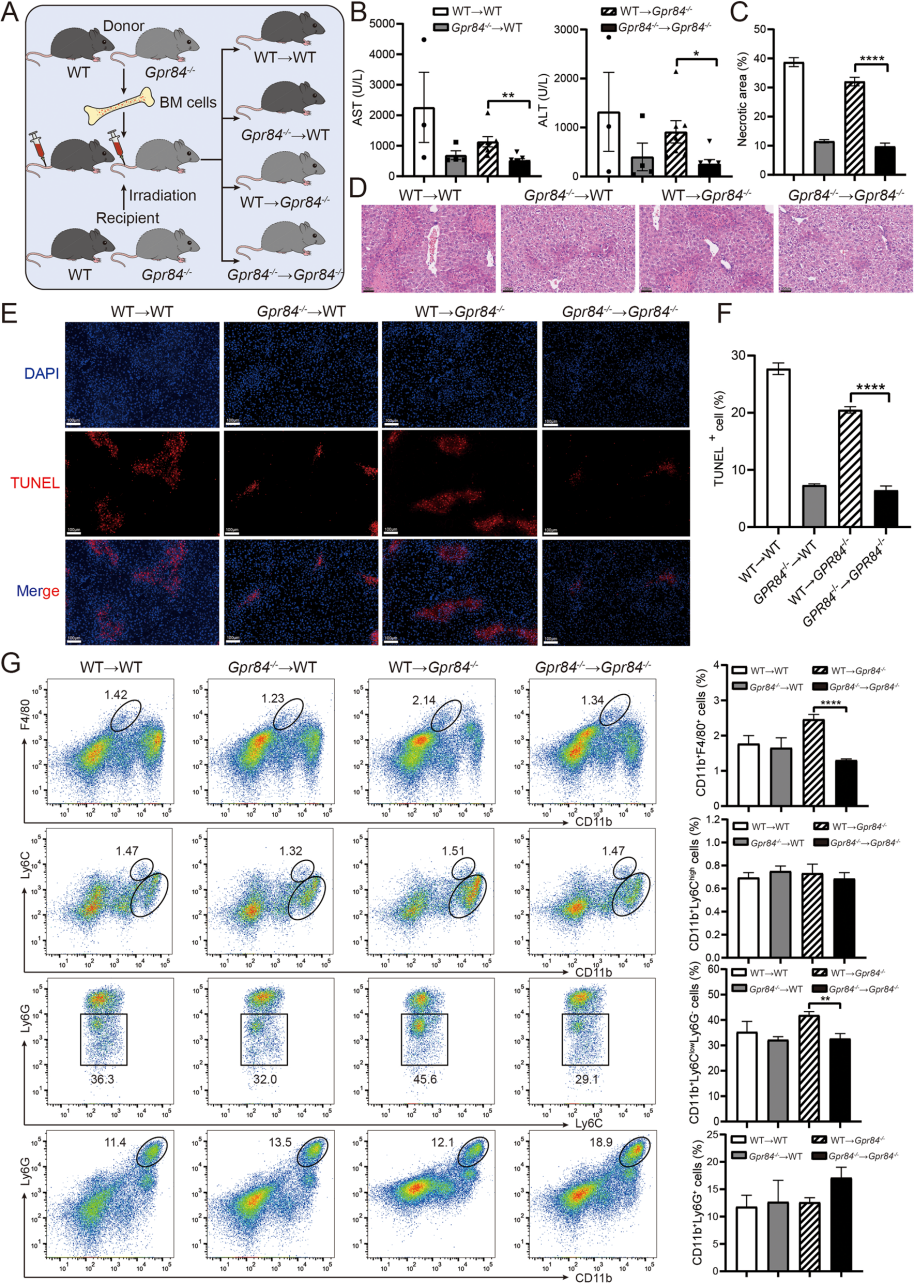
GPR84 expression on hematopoietic cells drives ConA-induced cell death in the liver. **A** Schematic of bone marrow transplantation to create chimeric mice: WT → WT (*n* = 4), *Gpr84*^*−/−*^ → WT (*n* = 4), WT → *Gpr84*^*−/−*^ (*n* = 7), and *Gpr84*^*−/−*^ → *Gpr84*^*−/−*^ (*n* = 7) chimeras. **B** Serum ALT and AST levels in chimeric mice after Con A injection. **C**, **D** H&E staining of liver tissue. **E**, **F** TUNEL staining of liver sections. **G** Flow cytometry analysis of NPC proportions in chimeric mice. Mean ± SEM. *P < 0.05, **P < 0.01, **** *P* < 0.0001

To further elucidate the role of GPR84-deficient immune cells in Con A-induced liver injury, we employed a bone marrow (BM) transplantation model to generate chimeric mice. This approach allowed us to investigate whether BM-derived cells lacking GPR84 are critical in mitigating Con A-induced hepatitis. Specifically, BM from *Gpr84*^*−/−*^ mice and WT mice was transplanted into irradiated *Gpr84*^*−/−*^ and WT recipients, respectively, generating four groups of chimeric mice: WT → WT, *Gpr84*^*−/−*^ → WT, WT → *Gpr84*^*−/−*^, and *Gpr84*^*−/−*^ → *Gpr84*^*−/−*^ (Fig. 4A). We then measured key indicators of liver injury following Con A administration. The results showed that the plasma levels of ALT and AST were significantly lower in the *Gpr84*^*−/−*^** → **Gpr84^*−/−*^ group compared to the WT → *Gpr84*^*−/−*^ group (Fig. 4B), suggesting that GPR84-deficient BM-derived cells attenuate Con A-induced liver injury. The results of H&E staining and TUNEL staining further confirmed that the extent of liver damage and the proportion of hepatocyte apoptosis in the *Gpr84*^*−/−*^ → *Gpr84*^*−/−*^ group were significantly lower than those in the WT → *Gpr84*^*−/−*^ group (Fig. 4C-F). Flow cytometry analysis of inflammatory cell infiltration further demonstrated that the proportion of Kupffer cells (CD11b^+^F4/80^+^) and infiltrating monocytes (CD11b^+^Ly6G^−^Ly6 C^low^, Ly6 C^lo^) was significantly lower in the *Gpr84*^*−/−*^ → *Gpr84*^*−/−*^ group than in the WT → *Gpr84*^*−/−*^ group (Fig. 4G). In contrast, there were no significant differences in the proportions of neutrophils (CD11b^+^Ly6G^+^) or infiltrating monocytes (CD11b^+^Ly6 C^high^, Ly6 C^hi^) between the groups. Similarly, no significant differences were observed in the ratios of CD4^+^ T cells or CD8^+^ T cells across the experimental groups (Fig. S1G). These findings suggest that GPR84-deficient hematopoietic cells, particularly macrophages, rather than liver parenchymal cells, play a pivotal role in Con A-induced immune hepatitis. This study highlights GPR84 as a crucial regulator of immune cell infiltration and inflammatory responses, offering novel insights into its role in immune modulation.

### GPR84 regulates macrophage secretion of inflammatory cytokines

**Fig. 5:**
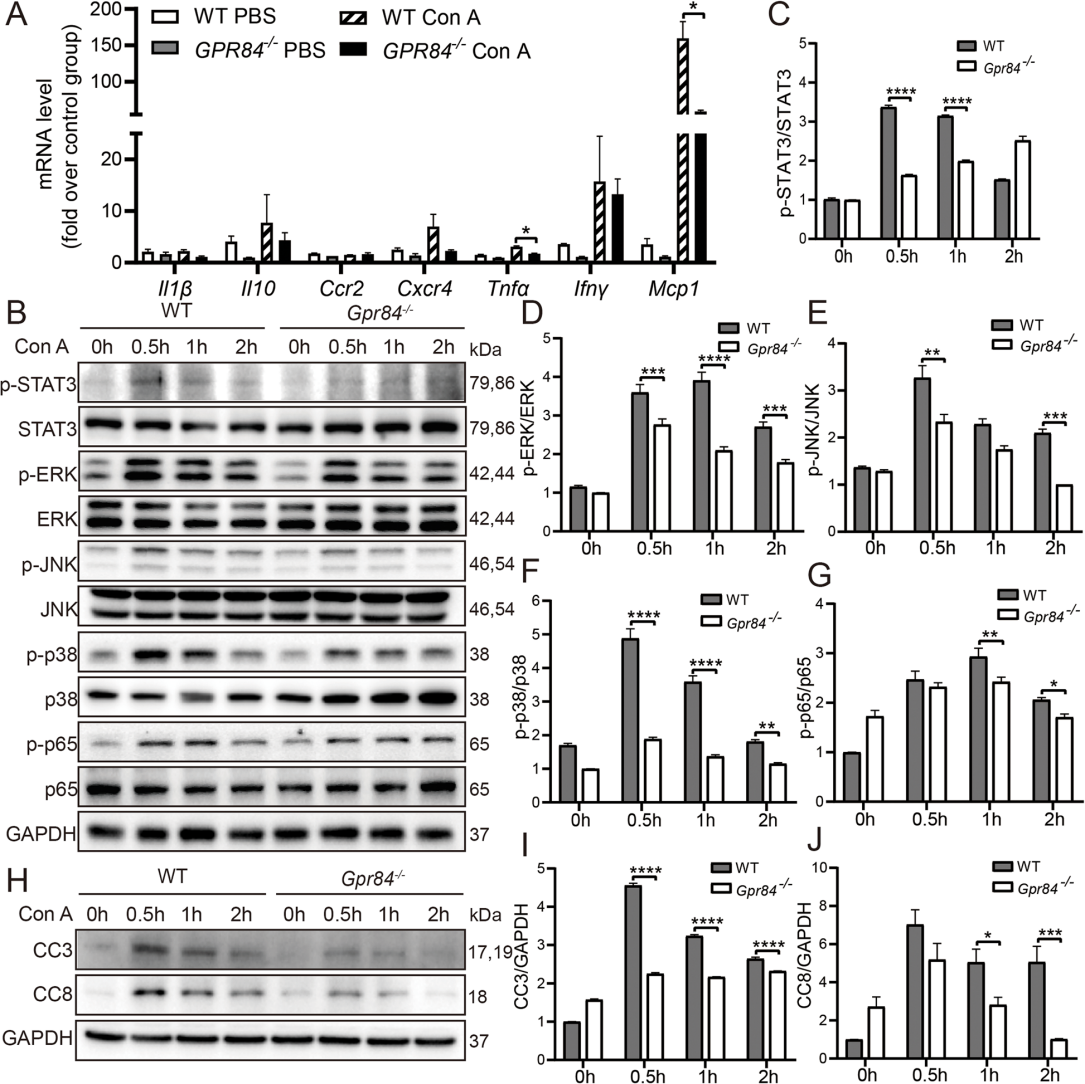
GPR84 regulates macrophage secretion of inflammatory cytokines. Bone marrow-derived macrophages (BMDMs) isolated from *Gpr84*^*−/−*^ and WT mice were treated with 10 μg/mL Con A for 12 h, (**A**) qRT-PCR analysis was used to assess the expression levels of inflammatory cytokines, (**B**–**G**) Western blotting was performed to evaluate the protein levels of STAT3, MAPK, and NF-κB signaling pathway components. **H**–**J** Primary hepatocytes were treated with supernatants from Con A-stimulated BMDMs, followed by Western blot analysis to measure the expression levels of Caspase-3 and Caspase-8. Mean ± SEM. *P < 0.05, **P < 0.01, *** *P* < 0.001, **** *P* < 0.0001

The above findings indicate that GPR84-deficient BM-derived cells, particularly macrophages, play a crucial role in alleviating Con A-induced immune liver injury. However, the precise impact of GPR84 on macrophage function requires further investigation. Therefore, we examined whether GPR84 deficiency influences macrophage functionality, particularly its regulation of inflammatory cytokine secretion. We isolated bone marrow-derived macrophages (BMDMs) from *Gpr84*^*−/−*^ and WT mice and treated them with 10 μg/mL Con A for 12 h. The expression levels of inflammatory cytokines were then analyzed using qPCR. The results showed that the expression levels of *Mcp1* and *Tnfα* were significantly lower in the *Gpr84*^*−/−*^ group compared to the WT group (Fig. 5A). To further explore the changes in signaling pathways, we treated BMDMs with Con A at different time points and assessed the activation of inflammation-related signaling pathways. The results demonstrated that the phosphorylation levels of p-STAT3, p-ERK, p-JNK, p-p38, and p-p65 were significantly higher in the WT group compared to the *Gpr84*^*−/−*^ group (Fig. 5B-G). Additionally, we collected the supernatants from Con A-treated BMDMs and used them to treat primary hepatocytes. By examining the expression of apoptosis-related proteins Caspase 3 and Caspase 8 in hepatocytes, we found that their levels were significantly lower in the *Gpr84*^*−/−*^ group compared to the WT group (Fig. 5H-J). In summary, GPR84-deficient macrophages produce fewer inflammatory cytokines, thereby alleviating Con A-induced immune liver injury. These results suggest that GPR84 plays a critical role in the onset and progression of immune liver injury by regulating macrophage inflammatory responses and signaling pathways.

### GLPG1205 alleviates Con A-induced immune liver injury in mice

**Fig. 6:**
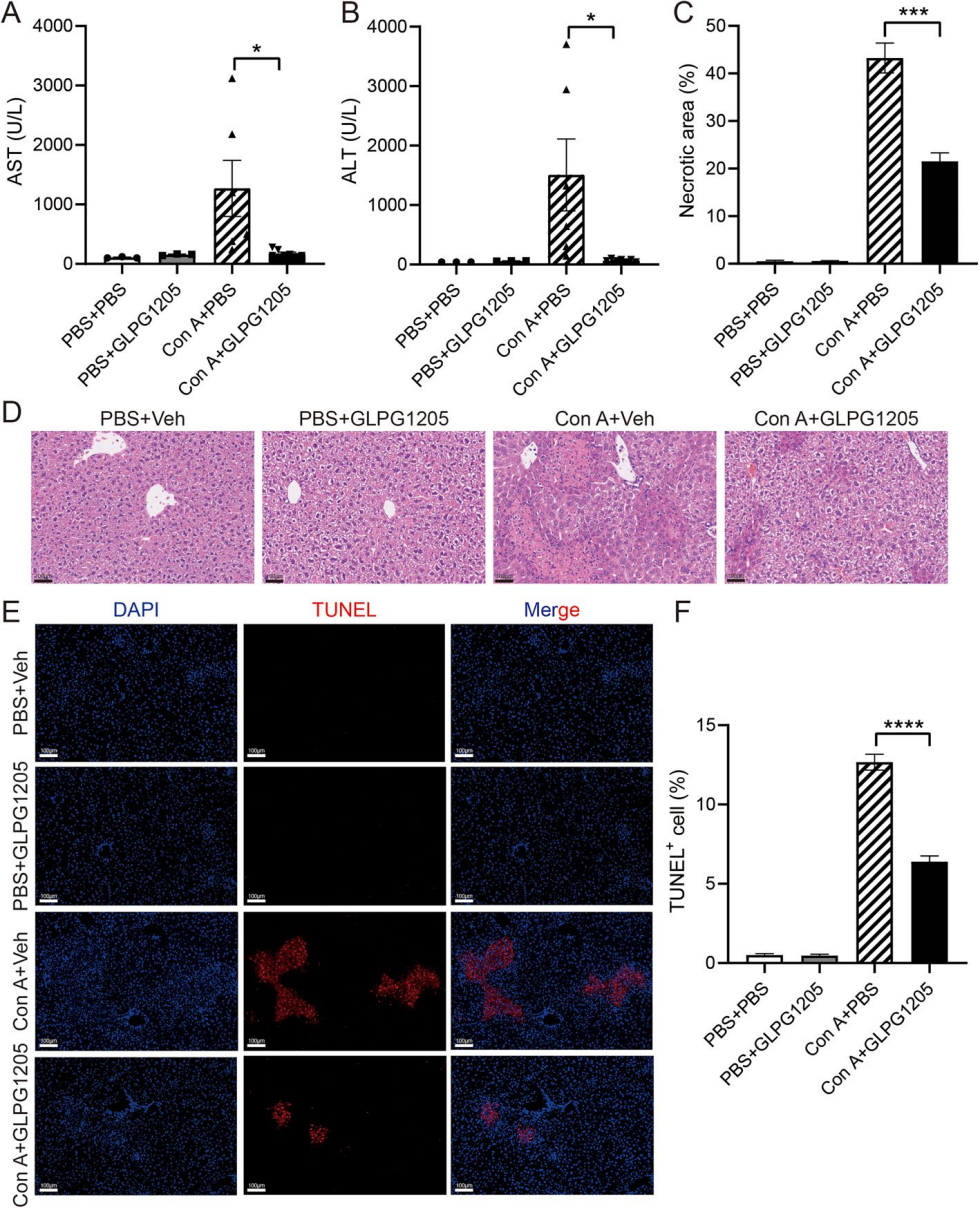
GLPG1205 alleviates Con A-induced immune liver injury in mice. WT mice were injected with Con A (15 mg/kg) for 1 h, treated with GLPG1205 or vehicle control by oral gavage, (**A**, **B**) Serum ALT and AST levels were measured. *n* = 3–7 mice per group. **C**, **D** H&E staining was performed to assess liver tissue damage. **E**, **F** TUNEL staining was used to evaluate the proportion of apoptotic hepatocytes. Mean ± SEM. *P < 0.05, *** *P* < 0.001, **** *P* < 0.0001

**Fig. 7:**
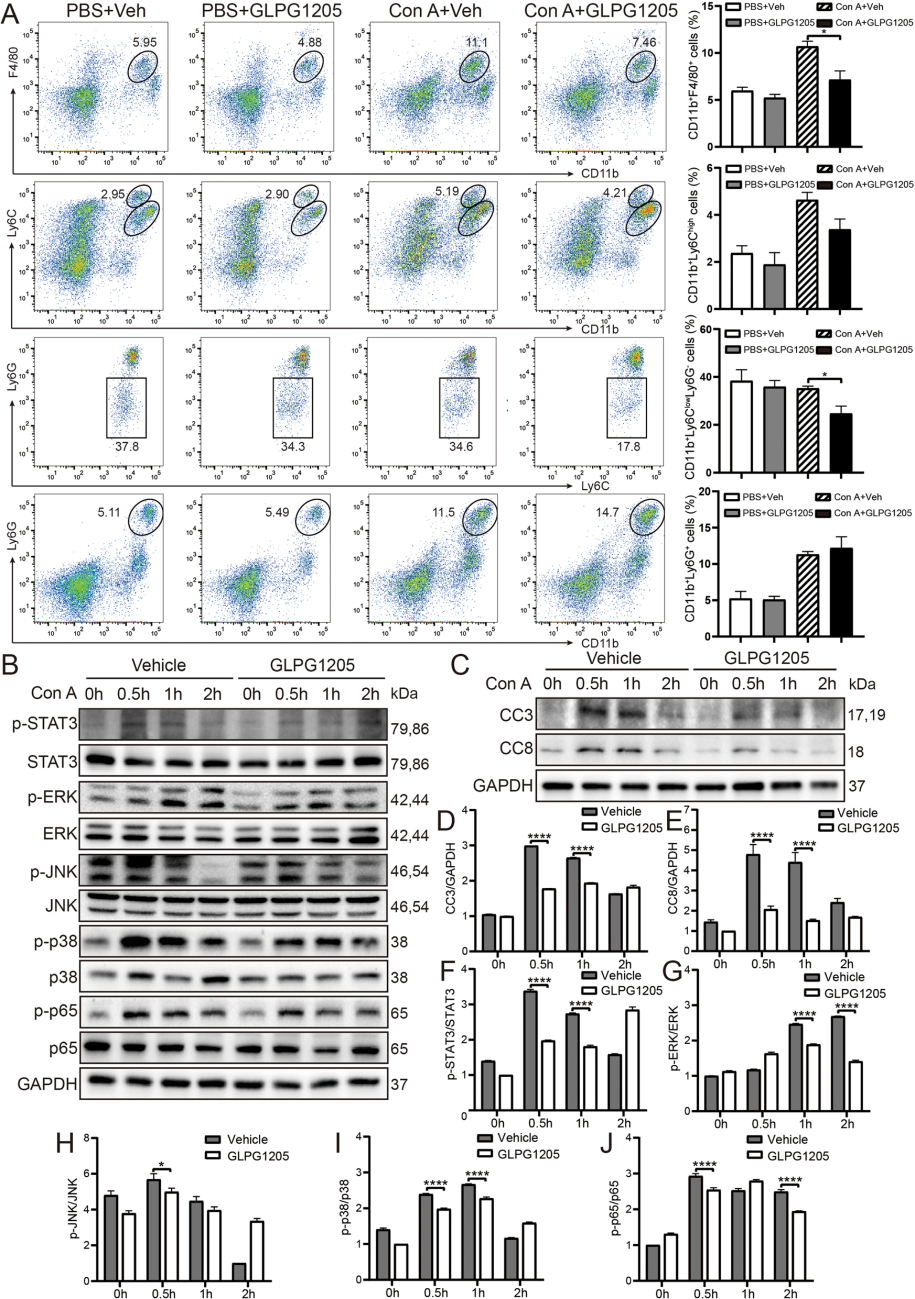
GLPG1205 reduces hepatocyte apoptosis by limiting inflammatory cell infiltration. WT mice were injected with Con A (15 mg/kg) for 1 h, and treated with GLPG1205 or vehicle control by oral gavage. **A** Flow cytometry was performed to analyze the proportions of non-parenchymal cells (NPCs) in the liver. **B**, **F**–**J** Bone marrow-derived macrophages (BMDMs) were pretreated with GLPG1205 for 1 h, followed by Con A treatment at 0 h, 0.5 h, 1 h, and 2 h. Western blotting was used to measure the expression of proteins involved in the STAT3, MAPK, and NF-κB signaling pathways. **C**–**E** Primary hepatocytes were treated with supernatants from Con A-stimulated BMDMs, and Western blotting was performed to detect the expression of apoptosis-related proteins, Caspase-3 and Caspase-8. Mean ± SEM. *P < 0.05, *** *P* < 0.001, **** *P* < 0.0001

**Fig. 8:**
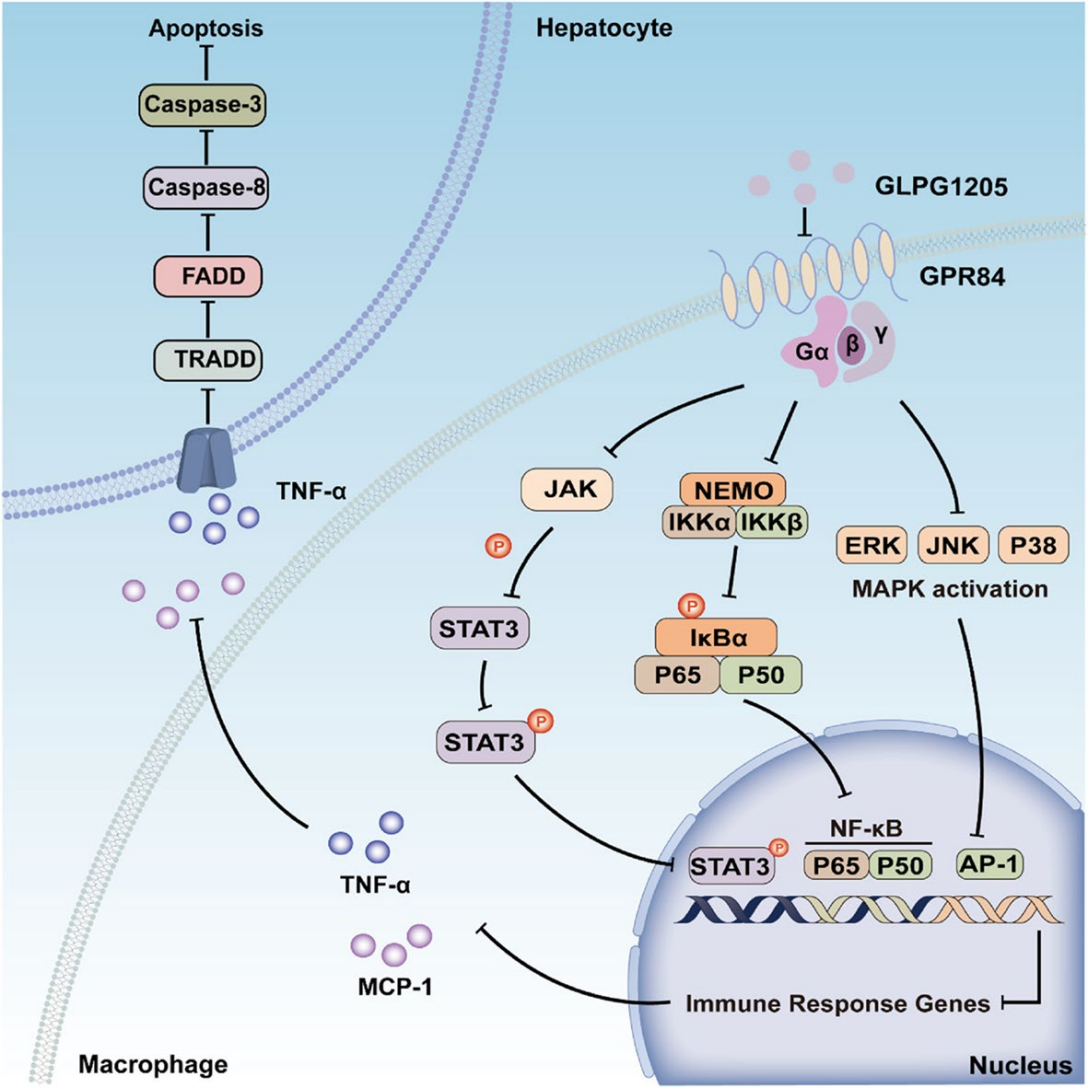
Schematic illustration of the protective effect of GLPG1205 against liver injury

Based on the above experimental results, the key role of GPR84 in immune liver injury was revealed, and the therapeutic effect of GPR84 antagonist GLPG1205 in a mouse model of immune liver injury induced by Con A was further explored. In vivo experiments, GLPG1205 treatment was performed 1 h after Con A induced liver injury, and the results showed that the serum ALT and AST levels in mice in the GLPG1205 treatment group were significantly reduced compared with the Vehicle treatment group (Fig. 6A, B), indicating that GLPG1205 effectively attenuated Con A-induced liver injury. H&E staining further confirmed that the liver injury area of mice in the GLPG1205 treatment group was significantly smaller than that in the Vehicle treatment group (Fig. 6C, D), and TUNEL staining showed that the proportion of hepatocyte apoptosis in the GLPG1205 treatment group was also significantly reduced (Fig. 6E, F). Next, we analyzed the inflammatory cell infiltration in each group of mice by flow cytometry. The results showed that the proportions of Kupffer cells (CD11b^+^F4/80^+^) and infiltrating monocytes (CD11b^+^Ly6 C^low^Ly6G^−^, Ly6 C^lo^) in the GLPG1205 treatment group were significantly lower than those in the Vehicle treatment group (Fig. 7A), while there was no significant difference in the proportions of neutrophils (CD11b^+^Ly6G^+^) and CD11b^+^Ly6 C^high^ infiltrating monocytes (Ly6 C^hi^) between the two groups. In addition, there was no significant difference in the ratio of CD4^+^ T cells and CD8^+^ T cells between groups (Fig.S1H). In vitro experiments, we pretreated bone marrow-derived macrophages (BMDMs) with GLPG1205 for 1 h, then stimulated the cells with Con A, and detected activation of inflammatory signaling pathways at different time points. The results showed that the expression levels of p-STAT3, p-ERK, p-JNK, p-p38 and p-p65 in the Vehicle group were significantly higher than those in the GLPG1205 treatment group (Fig. 7B, F-J). In addition, the expression levels of apoptosis-related proteins Caspase 3 and Caspase 8 were detected by treating primary hepatocytes with BMDMs supernatant after Con A. The results showed that the expression of Caspase 3 and Caspase 8 in the GLPG1205 treatment group was significantly lower than in the Vehicle treatment group (Fig. 7C-E). Overall, GPR84 antagonists GLPG1205 effectively alleviated Con A-induced immune liver injury in mice by reducing inflammatory cell infiltration and inhibiting the activation of inflammatory signaling pathways, showing its potential application prospects in the treatment of immune hepatitis (Fig. 8). Based on these findings, GPR84 modulation holds promise as a potential therapeutic strategy for the treatment of immune hepatitis. Future studies should further explore the clinical application of GPR84 inhibitors and evaluate their efficacy in patients with immune-mediated liver diseases. Based on these findings, modulating GPR84 presents a promising therapeutic strategy for the treatment of immune hepatitis. Future studies should further investigate the clinical application of GPR84 inhibitors and assess their efficacy in patients with immune-mediated liver diseases.

## Discussion

In recent years, there has been an increasing number of studies on the role of GPR84 in liver-related diseases, especially in chronic diseases such as fatty liver and liver fibrosis (Ohue-Kitano et al. 2023; Puengel et al. 2020). Puengel et al. found that in nonalcoholic fatty liver disease (NAFLD), GPR84 expression was upregulated and correlated with the severity of inflammation and fibrosis (Puengel et al. 2020). In this study, we discovered that GPR84 deficiency significantly improved immune-mediated liver injury in mice subjected to Con A treatment, as reflected by reduced transaminase levels and smaller areas of liver damage. These findings suggest that the impact of GPR84 on liver injury may involve both metabolic and immune mechanisms.

Previous studies have indicated that T cells, particularly CD4^+^ T cells, play a central role in the pathogenesis of Con A-induced immune liver injury (Tiegs et al. 1992; Ye et al. 2018). In addition, Con A treatment promotes the recruitment of neutrophils, which intensifies inflammation at the site of liver injury (Mathews et al. 2016). Kupffer cells, the liver’s resident macrophages, also play a pivotal role by releasing inflammatory cytokines upon activation (Yang et al. 2013). Our research revealed that GPR84 deficiency mainly reduced the infiltration of macrophages and infiltrating monocytes, while the infiltration of T cells remained unaffected. Moreover, inflammatory cytokine analysis showed that the expression levels of *Mcp1* and *Tnfα* were significantly lower in GPR84-deficient mice, indicating that GPR84 modulates the inflammatory response. Notably, *Gpr84*^*−/−*^ mice that received BM transplants from either WT or *Gpr84*^*−/−*^ donors displayed distinct pathological outcomes under Con A-induced liver injury. This suggests that GPR84 plays an essential role not only in immune cells but also in non-hematopoietic cells, highlighting its dual role in both immune regulation and liver function.

Con A-induced immune liver injury involves multiple inflammatory signaling pathways, such as STAT3, JNK, AKT, ERK, and p38 MAPK. Activation of these pathways exacerbates inflammation and cell apoptosis, worsening liver injury (Tiegs et al. 1992; Ye et al. 2018; Mathews et al. 2016; Yang et al. 2013). Our study revealed similar results: these pathways exhibited greater activation in WT mice than in *Gpr84*^−/−^ mice. This suggests that GPR84 deficiency effectively suppressed their activation, thereby mitigating liver injury.

Given the role of GPR84 in inflammatory diseases, several GPR84 antagonists, including GLPG1205, PBI-4050, and PBI-4547, have been developed to explore their therapeutic potential in models of inflammation and fibrosis (Chen et al. 2020). Notably, PBI-4050 and PBI-4547 are also GPR40 agonists (Gagnon et al. 2018; Simard et al. 2020), which led us to focus on GLPG1205 for assessing its therapeutic effect on Con A-induced immune-mediated liver injury. Although GLPG1205 did not demonstrate significant efficacy in Phase II clinical trials for ulcerative colitis and idiopathic pulmonary fibrosis (IPF) (Strambu et al. 2023; Labéguère et al. 2020), it exhibited promising therapeutic effects in our Con A-induced liver injury model. The beneficial effects of GLPG1205 were primarily achieved by suppressing macrophage and infiltrating monocyte secretion of inflammatory cytokines, thereby reducing liver injury.

Our findings provide new insights into the broad role of GPR84 in immune-mediated liver diseases, suggesting that targeting GPR84 could be a promising strategy for treating acute immune-mediated liver injury with potential therapeutic value. This discovery aligns with previous studies on the role of GPR84 in liver inflammation and fibrosis, further highlighting its potential as a therapeutic target in inflammatory diseases. Although the study results are promising, several limitations should be considered. First, the specificity of GPR84 inhibitors remains a challenge, as potential off-target effects may influence the observed outcomes. Although GLPG1205 demonstrated favorable efficacy in our research model, the translational process from experimental studies to clinical application may be lengthy, requiring further evaluation of its clinical feasibility.

In conclusion, our study demonstrates that GPR84 plays a key role in exacerbating Con A-induced immune liver injury by regulating the inflammatory response of macrophages and infiltrating monocytes. Importantly, the GPR84 antagonist GLPG1205 significantly alleviated liver injury, highlighting GPR84 as a promising therapeutic target for immune-mediated liver diseases. Given the clinical relevance of our findings, future studies should focus on further validating the efficacy and safety of GPR84 inhibitors in preclinical models and exploring their potential for clinical trials in autoimmune hepatitis.

## Materials and methods

### Acquisition and analysis of public data resources

We retrieved the dataset for Con A-induced immune liver injury in mouse models (GSE17184, n = 12) from the Gene Expression Omnibus (GEO) database. Using the SangerBox platform (http://sangerbox.com), we identified differentially expressed genes (DEGs) and generated relevant visualizations.

### Animals

The animal experiments were conducted using 6–8-week-old male C57BL/6 J wild-type (WT) mice, obtained from the Model Animal Research Center of Nanjing University. GPR84 knockout (KO) mice were provided by BRL Medicine Inc. Mice were housed under controlled temperature conditions with a standard 12-h light/dark cycle. All animal procedures were approved by the Animal Experimentation Center of Nanjing Medical University.

### Induction of immune liver injury

WT and GPR84-deficient mice were subjected to immune liver injury by tail vein injection of Concanavalin A (Con A, 15 mg/kg, Sigma, #C2010) or phosphate-buffered saline (PBS). Mice were euthanized at different time points post-injection via CO₂ inhalation to collect liver tissues and blood samples for subsequent analyses.

### Quantitative real-time PCR (qRT-PCR)

Total RNA was extracted from liver tissues using Trizol reagent, followed by reverse transcription into cDNA with HiScript III RT SuperMix (Vazyme, #R323). The cDNA was then amplified using ChamQ SYBR Color qPCR Master Mix (Vazyme, #Q421). Primers used in the PCR reactions were synthesized by Invitrogen, and their sequences are listed in Supplementary Table 1.

### Immunohistochemistry (IHC)

Mouse liver tissues were fixed in 4% paraformaldehyde for 24 h, followed by paraffin embedding and sectioning. The sections were stained with H&E and subjected to immunohistochemical staining using a GPR84 antibody (Bioss, #bs-13507R). Stained sections were examined and photographed with an Olympus IX51 light microscope, and ImageJ software was used to analyze tissue morphology.

### Western blotting

Total protein was extracted from mouse liver tissues or cell samples using RIPA lysis buffer. Protein samples were separated by SDS-PAGE and transferred to PVDF membranes. After blocking with 5% BSA for 2 h, membranes were incubated overnight at 4 °C with primary antibodies, followed by detection with HRP-conjugated secondary antibodies. The primary antibodies used included p-STAT3 (Cell Signaling Technology, #4074S), STAT3 (Cell Signaling Technology, #4904S), p-ERK (Cell Signaling Technology, #4370S), ERK (Cell Signaling Technology, #4695S), p-JNK (Cell Signaling Technology, #9251S), JNK (Cell Signaling Technology, #9252S), p-p38 (Cell Signaling Technology, #9211S), p38 (Cell Signaling Technology, #9212S), p-p65 (Cell Signaling Technology, #3033S), p65 (Cell Signaling Technology, #8242S), Cleaved Caspase-8 (Cell Signaling Technology, #8592S), Caspase-3/p17/p19 (Proteintech, #66,470–2-Ig), GPR84 (Bioss, #bs-13507R), and GAPDH (Proteintech, #10,494–1-AP). All antibodies were diluted according to the manufacturer’s instructions.

### AST and ALT measurement

Blood samples were collected from the retro-orbital sinus of mice at 8 and 24 h after Con A injection. Serum was separated and analyzed using an automated biochemical analyzer (MODULAR EVO 4200) to measure AST and ALT levels.

### TUNEL assay

To detect apoptosis, the One Step TUNEL Apoptosis Assay Kit (Beyotime, #C1089) was used according to the manufacturer's instructions for TUNEL staining.

### Liver non-parenchymal cells (NPCs) isolation

The liver was perfused with HBSS containing EGTA to remove blood cells, followed by mechanical dissociation. The tissue was ground, filtered through a 100 µm cell strainer to obtain a single-cell suspension, and red blood cells were lysed using RBC lysis buffer. Liver NPCs were then isolated using 35% Percoll (Cytiva, #17,089,101). The resulting cells were resuspended in PBS with 2% FBS for subsequent staining.

### Flow cytometry

Isolated liver NPCs were incubated with CD16/32 antibody for 30 min to block non-specific binding, followed by staining with fluorescence-conjugated antibodies. The antibodies used included: APC-conjugated anti-mouse F4/80 (BioLegend, #123,116), FITC-conjugated anti-mouse Ly6 C (BD Biosciences, #561,085), PE-Cyanine7 conjugated anti-mouse CD11b (BioLegend, #101,216), APC-Cyanine7 conjugated anti-mouse CD45 (BioLegend, #103,116), PE-conjugated anti-mouse Ly6G (BD Biosciences, #561,104), APC-eFluor™ 780-conjugated anti-mouse CD3 (eBioscience, #47–0032–80), APC-conjugated anti-mouse CD4 (eBioscience, #17–0042–83), and FITC-conjugated anti-mouse CD8a (eBioscience, #11–0081-81).

### Isolation and culture of bone marrow-derived macrophages (BMDMs)

Mice were euthanized with CO₂, and bone marrow was flushed from femurs using RPMI 1640 medium. The cell suspension was passed through a 100 µm strainer, and red blood cells were removed with lysis buffer. The remaining cells were seeded in culture dishes with RPMI 1640 medium supplemented with 10 ng/mL macrophage colony-stimulating factor (M-CSF) and 10% FBS. After incubating at 37 °C with 5% CO₂ for 4 days, 4 mL of fresh medium containing 10 ng/mL M-CSF and 10% FBS was added. Cells were incubated for an additional 3 days before being used for further experiments.

### Isolation of primary hepatocytes

Primary hepatocytes were isolated following the protocol (You et al. 2013). In brief, after euthanasia, the liver was first perfused with HBSS containing Ca^2^⁺ and Mg^2^⁺ for 5 min, followed by 2 min with HBSS without Ca^2^⁺ and Mg^2^⁺. Subsequently, 0.04% type IV collagenase (Sigma-Aldrich, #C4-BIOC) was perfused for 10 min. The liver was then gently stirred in Williams E medium and filtered through a 100 µm cell strainer. Hepatocytes were isolated by centrifugation at 40 × g for 3 min.

### Bone marrow transplantation to create chimeric mice

Eight-week-old recipient mice were fed 0.2% neomycin for one week prior to X-ray irradiation (5 Gy for 2–3 min, repeated after 3 h). Donor bone marrow cells (1 × 10^7^ cells/mouse) were injected via the tail vein immediately after irradiation. The transplanted mice were housed in cages and maintained on 0.2% neomycin for two weeks to prevent infections. Eight weeks after transplantation, immune-mediated liver injury was induced by injecting Con A (15 mg/kg, Sigma, #C2010). To confirm bone marrow reconstitution, genomic DNA was extracted from peripheral blood and used for GPR84 genotyping (Fig. S1D).

### Treatment

To induce immune-mediated liver injury, mice received an injection of Con A (15 mg/kg, Sigma, #C2010) or PBS. One hour later, 30 mg/kg of the GPR84 antagonist GLPG1205 (MCE, HY-135303) was administered via oral gavage. Mice were euthanized with CO₂, and liver tissues and blood samples were collected for further analysis. For cell experiments, cells were treated with 10 µM GLPG1205 and 10 µg/mL Con A, and samples were collected at various time points for downstream protein analysis.

### Statistical analysis

All data are presented as the mean ± SEM. Statistical analyses were performed using GraphPad Prism software (version 8.0.2). Depending on the number of groups compared, either a two-tailed unpaired Student’s *t*-test or analysis of variance (ANOVA) was used to assess statistical significance. For comparisons between two groups, we utilized the Student's t-test, employing the Mann–Whitney U test for non-parametric data. For comparisons involving multiple groups, we conducted one-way or two-way analysis of variance (ANOVA), followed by post-hoc tests to identify specific differences among groups. Statistical significance was indicated by *P* values, with *P* < 0.05 considered significant. Levels of significance are denoted as P < 0.01 (**), P < 0.001 (***), and P < 0.0001 (****).

## Supplementary Information

Supplementary Material 1.

## Data Availability

No datasets were generated or analysed during the current study.
