# Supplementary figures and images for: Targeting GPR84 to alleviate acute immune-mediated liver injury
Source: Mol Med. 2025 May 14;31:187. doi: 10.1186/s10020-025-01248-9 (PMC12080032; doi:10.1186/s10020-025-01248-9)

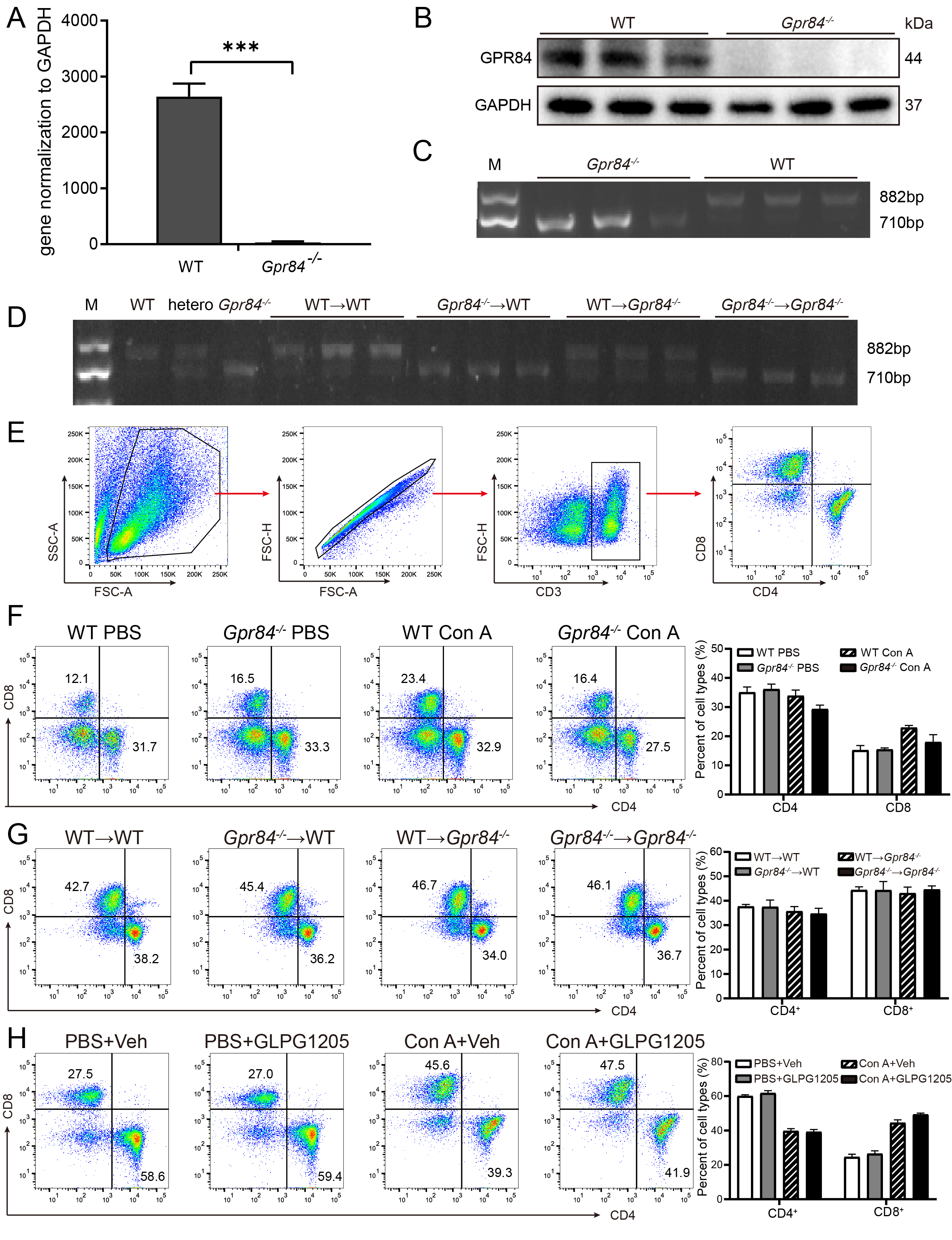

Supplement: Supplementary file 1 — Supplementary Material 1. [file 10020_2025_1248_MOESM1_ESM.jpg]
